# Quantitative Renal Perfusion Measurements in a Rat Model of Acute Kidney Injury at 3T: Testing Inter- and Intramethodical Significance of ASL and DCE-MRI

**DOI:** 10.1371/journal.pone.0053849

**Published:** 2013-01-07

**Authors:** Fabian Zimmer, Frank G. Zöllner, Simone Hoeger, Sarah Klotz, Charalambos Tsagogiorgas, Bernhard K. Krämer, Lothar R. Schad

**Affiliations:** 1 Computer Assisted Clinical Medicine, Medical Faculty Mannheim, Heidelberg University, Mannheim, Germany; 2 Department of Medicine V, University Medical Centre Mannheim, Heidelberg University, Mannheim, Germany; 3 Clinic for Anaesthesiology and Intensive Care, University Medical Centre Mannheim, Heidelberg University, Mannheim, Germany; The University of Manchester, United Kingdom

## Abstract

**Objectives:**

To establish arterial spin labelling (ASL) for quantitative renal perfusion measurements in a rat model at 3 Tesla and to test the diagnostic significance of ASL and dynamic contrast-enhanced magnetic resonance imaging (DCE-MRI) in a model of acute kidney injury (AKI).

**Material and Methods:**

ASL and DCE-MRI were consecutively employed on six Lewis rats, five of which had a unilateral ischaemic AKI. All measurements in this study were performed on a 3 Tesla MR scanner using a FAIR True-FISP approach and a TWIST sequence for ASL and DCE-MRI, respectively. Perfusion maps were calculated for both methods and the cortical perfusion of healthy and diseased kidneys was inter- and intramethodically compared using a region-of-interest based analysis.

**Results/Significance:**

Both methods produce significantly different values for the healthy and the diseased kidneys (P<0.01). The mean difference was 147±47 ml/100 g/min and 141±46 ml/100 g/min for ASL and DCE-MRI, respectively. ASL measurements yielded a mean cortical perfusion of 416±124 ml/100 g/min for the healthy and 316±102 ml/100 g/min for the diseased kidneys. The DCE-MRI values were systematically higher and the mean cortical renal blood flow (RBF) was found to be 542±85 ml/100 g/min (healthy) and 407±119 ml/100 g/min (AKI).

**Conclusion:**

Both methods are equally able to detect abnormal perfusion in diseased (AKI) kidneys. This shows that ASL is a capable alternative to DCE-MRI regarding the detection of abnormal renal blood flow. Regarding absolute perfusion values, nontrivial differences and variations remain when comparing the two methods.

## Introduction

Most renal and renovascular diseases like renal artery stenosis or renal insufficiency are initially without any symptoms or pain. Therefore, the importance of radiation free methods which allow the assessment of renal function by measuring renal microvascular perfusion is crucial to diagnose and to treat renal diseases already in early stages of their progression.

Magnetic resonance imaging (MRI) not only provides excellent morphological information due to the different T_1_ and T_2_ relaxation times of different kidney tissues [1], but also high enough spatial and temporal resolution for a functional analysis of the kidneys [2]. For the assessment of renal perfusion data with MRI, two techniques exist: Dynamic contrast-enhanced (DCE)-MRI and arterial spin labelling (ASL). DCE-MRI involves the injection of a contrast agent, usually gadolinium (Gd) based, as a tracer and allows to assess renal blood flow (RBF) and glomerular filtration rate (GFR) by applying different physiological models [3]–[5]. However, in the last years, cases of nephrogenic systemic fibrosis (NSF) associated with gadolinium based contrast agents have been reported [6]. ASL does not have this disadvantage as it is a completely non-invasive method that uses magnetically labelled water protons in arterial blood as an endogenous tracer [7], [8]. This way, by combining one of the three main labelling schemes (pulsed labelling (PASL) [8]–[11], continuous labelling (CASL) [12] or pseudo-continuous labelling (pCASL) [13]) with an adequate imaging sequence, a perfusion sensitive experiment can be designed. As no exogenous tracer is used, it is a technique that can be repeatedly employed on the same patient within short time intervals. Originally, spin labelling techniques including selective labelling pulses [14] were only used to assess cerebral perfusion. However, with the advent of fast imaging sequences that are less susceptible to field inhomogeneities, applications in the abdomen became possible. Due to their high perfusion, the kidneys have been one of the first sites where ASL was applied outside the brain and several studies have shown the feasibility of quantitative renal perfusion for healthy, diseased and transplanted kidneys [15]–[20].

Although there are several approaches and models [9], [10], [21]–[25] to quantitate blood flow with ASL, there are a number of challenges and aggravating issues, especially in the abdomen. Problems comprise movement from respiratory-related motion and commonly a low perfusion signal of only a few percent. Other problematic issues concerning the quantitation are the transit times of blood from the labelling region to the imaging slab, the exchange time of blood from arteries into the tissue and the blood-tissue partition coefficient. Although there exist different approaches to overcome some of the mentioned issues [20], [26], the value and the validity of ASL have to be tested. Artz et al. [27] have validated ASL perfusion estimates in an interventional swine model by comparing it against fluorescent microsphere measurements. However, ASL also needs to be tested in diseased kidneys and the estimates have to be compared to an alternative MR perfusion measurement, i.e. DCE-MRI. DCE-MRI is more commonly used in clinical routine and enjoys a higher reputation amongst physicians compared to ASL. Meaningful ASL measurements might be especially valuable in cases were the injection of contrast agent is contraindicated, e.g. when renal diseases are already known or when possible interactions between drugs and contrast agent might invalidate drug studies. In particular, studies examining the effect of drugs to treat renal diseases or to protect transplanted kidneys [28], [29] could benefit from a non-invasive measurement with ASL.

The objective of this study was to establish ASL for renal perfusion measurements in rats at a 3 Tesla human scanner and to investigate and compare its significance against that of DCE-MRI. Although, both methods used in this study have already progressed to clinical research they are still not well established for renal perfusion measurements and no consensus on the used imaging strategies has been reached [30], [31]. Furthermore, no renal diseases have been examined in clinical research by using both DCE-MRI and ASL. Therefore, an animal study performed on a whole-body scanner has the advantage that dedicated renal diseases can be investigated and the used methods and gained knowledge can easily be transferred to clinical research or even routine.

In this study, both methods were consecutively employed to measure the renal perfusion of rats with unilateral ischaemic acute kidney injury (AKI). We used a well-standardised warm ischaemia model that decreases perfusion significantly [32], [33] as a consequence of microvascular damage and interstitial edema, resulting in functional deterioration of the kidney [34]. With the acquired data, maps of RBF were calculated by using a simple model based on modified Bloch equations and a deconvolution approach, respectively. Finally, a region of interest (ROI) based evaluation of the perfusion maps was performed to assess and to evaluate the estimates of renal cortical perfusion.

As perfusion measurements with contrast agents also face challenges, e.g. the proper choice of the arterial input function (AIF), this was not supposed to be a comparison against DCE-MRI as a perfusion-‘gold standard’ but rather the comparison to one commonly used alternative when measuring perfusion with MRI. Hereby, the major focus was put on the ability to discriminate healthy and diseased kidneys while the comparison of absolute perfusion values is only secondary.

## Materials and Methods

All measurements were performed on a 3T whole-body MR scanner (Magnetom Tim Trio, Siemens Healthcare Sector, Erlangen, Germany) operating at a maximum gradient strength of 45 mT/m and a maximum slew rate of 200 T/m/s. We used an eight channel receive-only volumetric rat array (RAPID Biomedical GmbH, Rimpar, Germany) for signal detection. The body coil was used for homogeneous RF transmission.

A total of six Lewis rats were examined in this study, five of which had an ischaemic acute kidney injury (AKI). The animals were placed supine and head first so that the kidneys were in the RF centre of the rat array. To ensure sufficient coupling of the rat to the body coil, the transmitter voltage was adjusted manually at the beginning of each experiment.

### Animals

Six male Lewis (LEW, RTI1) rats with a weight of 260 to 290 g were obtained from Charles River (Sulzfeld, Germany). Animals were kept under standard conditions. They were fed with standard rodent chow and water ad libitum.

#### Ethics

All procedures were performed according to the Guide of the Care and Use of Laboratory Animals published by the National Academy of Sciences and were approved by the local authorities (Regional council Karlsruhe, G40/10).

#### Warm ischaemia model

Acute kidney injury (AKI) is a common clinical problem and might be caused by ischaemia-reperfusion injury (IRI) [35]. The warm ischaemia model is a well-standardised animal model which is used to induce IRI. For that purpose the renal artery and renal vein are clamped for an ischaemia time of 45 min [34]. Because of using nontraumatic clamps, renal artery and renal vein do not show any modifications afterwards. Ischaemia and therefore hypoxia lead to endothelial lesion and damage of the barrier function in the kidney resulting in interstitial edema. Furthermore, renal structures with a high demand of oxygen like tubular epithelial cells are strongly impaired during ischaemia showing tubulus dilatation and necrosis [36]. Renal perfusion in AKI decreases up to 50% [33] as a consequence of microvascular damage and interstitial edema, resulting in functional deterioration of the kidney.

#### Animal preparation

Five animals were subjected to acute kidney injury, one native rat served as control to optimise the MRI measurements at the beginning. Before laparotomy the rats were anaesthetized with ketamine (Ketamin 10%®, Intervet GmbH, Unterschleißheim, Germany) and xylazine (Rompun 2%®, Bayer Vital GmbH, Leverkusen Germany) and were heparinized (100 IE, Heparin-Natrium ratiopharm®, Ratiopharm GmbH, Ulm, Germany) before clamping the renal artery and the renal vein of the left kidney for 45 min. While opening the clamps the renal reperfusion was evaluated macroscopically. The native right kidney served as direct control. During surgery, a hotplate (38°C) was used to keep the animals’ body temperature in a normal range. Postoperative, the animals were exposed to an infrared light until they were fully awakened. All efforts were made to minimise suffering including a unique analgesic treatment with buprenorphinhydrochlorid and a daily follow-up until the experiment.

### MRI

The experiments were conducted five days after the induction of the AKI to ensure a significant histologic damage. Ten minutes prior to the MRI measurements animals were anaesthetised with thiobutabarbital sodium (Inactin®, SIGMA-Aldrich Chemie GmbH, Steinheim, Germany) and were transported to the MR scanner in a specific isolated transportation box. Before starting the MRI measurements a catheter was inserted in the right vena femoralis for the application of the contrast agent (Dotarem®, Guerbet, Roissy CdG Cedex, France) during the DCE-MRI. The body temperature of the rats was not monitored during MRI.

#### Arterial spin labelling

ASL measurements were carried out using the combination of a FAIR labelling scheme (PASL) and a true fast imaging with steady-state precession (True-FISP) sequence [37]. We imaged a single axial slice with a thickness of 4 mm. The slice was positioned in such a way that both left and right kidney were imaged at the same time. Images without magnetic preparation (M_0_), with global inversion (ns-IR, tag) and with slice-selective inversion (ss-IR, control) were recorded in an interleaved manner. Overall, 90 images were acquired resulting in 30 tag-control pairs and 30 M_0_ images to assess the signal intensities. For both global and slice-selective inversion an adiabatic hyperbolic secant pulse [38] was used which was VERSE [39] transformed in the case of slice-selective inversion to reduce SAR. The inflow time (inversion time) TI was 1.2 s in both (ss-IR and ns-IR) cases. The slice thickness for the selective inversion was 8 mm. Its slab was orientated and located so that it covered the imaging volume completely and symmetrically. This ensured a complete inversion of the imaging slab and strongly decreased the risk of a displacement of the imaging slab outside the inversion volume during the respiratory cycle. The profile quality of the ss-IR inversion was verified in a water phantom.

Echo time (TE) for the True-FISP was 2.72 ms, repetition time (TR) was 5.44 ms, bandwidth was 651 Hz/pixel and the flip angle was 70°. We used a 256×256 imaging matrix and a 140×140 mm^2^ field of view which resulted in an in-plane resolution of 0.5×0.5 mm^2^. The images were recorded in 0.6 s by using a PAT acceleration factor of 3. The inter image time was 6 s to ensure full longitudinal relaxation and an equal initial condition for each labelling mode. To achieve a high sensitivity to perfusion centric reordered phase-encoding was used. The image quality was improved by using an α/2 preparation pulse and 10 dummy pulses before the signal recording started [19]. The total measurement time was 9 min.

For rat 2 and rat 4 four consecutive measurements were performed to test the within subject variability.

#### DCE-MRI

Immediately after the ASL imaging dynamic contrast enhanced (DCE) perfusion imaging was performed. Therefore, we used a 3D time-resolved angiography with stochastic trajectories (TWIST) sequence [40] with the following parameters: TR = 3.4 ms, TE = 1.4 ms, a flip angle of 20°, field of view 114×50 mm^2^, and generalised autocalibrating partially parallel acquisition (GRAPPA) of factor 2. Matrix size was 192×84×28 and voxel resolution was 0.6×0.6×1.2 mm^3^. TWIST view sharing was set to 15% central region and 20% sampling density in the outer region. The nominal temporal resolution was 0.9 s per volume. Images were continuously acquired for 6 minutes. According to a normal clinical dose and the manufacturer instruction about 0.05 ml of contrast agent (Dotarem®, Guerbet, Roissy CdG Cedex, France), followed by a saline flush of 1 ml, was administered manually after the 15th volume was acquired.

#### Data evaluation

ASL perfusion maps were calculated by analysing the acquired data with an in-house written MATLAB (Version 7.10, The MathWorks, Natick, MA) script. After the images were averaged according to their magnetic preparation (ns-IR, ss-IR and M_0_) the perfusion-weighted difference image ΔM between the average ss-IR and the average ns-IR image was calculated. A registration of the images to assure a proper collocation was not required as no in-plane motion in the transversal images was visible and measureable. We followed the approach of the signal equation difference between the ss-IR and the ns-IR [9], [10] and thereby, neglected transit effects. We assumed an immediate exchange of blood protons into the tissue. Using the calculated ΔM image at time TI = 1.2 s and the average M_0_ image, perfusion maps (f) were then calculated on a pixel-by-pixel basis according to the following equation [9], [19]:
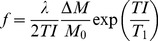
(1)


Here, λ is the blood-tissue water partition coefficient and was assumed to have a constant value of 0.8 ml/g [17]. T_1_ is the longitudinal relaxation time of the tissue. We used T_1_ = 1.14 s as proposed by de Bazelaire et al. [1]. To assess the mean perfusion rate in the renal cortex, a region of interest (ROI) was drawn in the perfusion maps delineating the renal cortex. For left and right kidney of each rat, the mean cortical blood flow of all pixels inside the ROI was estimated.

Quantification of DCE-MRI kidney perfusion was performed using a pixel-by-pixel deconvolution approach using the following equation:
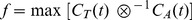
(2)


Here, C_T_(t) and C_A_(t) are the contrast concentrations as a function of time in a region of interest in the tissue and inside the artery feeding the region of interest (arterial input function), respectively. The renal perfusion *f* then is the maximum value of the tissue impuls response function, i.e. the deconvolution of the two concentration functions. The quantification was implemented in an in-house OsiriX plug-in [41]. The software calculates maps of the RBF. The arterial input function was determined by carefully placing a ROI in the abdominal aorta cranial to the branch of the renal arteries to avoid inflow effects and to minimize partial volume effects due to the small vessel diameter. All data were normalised by subtracting the mean intensity of 15 baseline volumes, and a linear relationship of the contrast agent concentration to the measured signal intensities was assumed. Only the volumes capturing solely the first pass were taken for calculating the perfusion parameters, i.e. 60 volumes for animal three and 50 volumes for all others. This way, errors caused by the clearance of contrast agent through the medullary pyramids were excluded.

To allow for a comparison, the ROI to assess the RBF from the DCE perfusion maps was drawn in consensus with the ROI in the ASL evaluation. For DCE-MRI, the mean value out of the three slices corresponding to the ASL slice was taken.

A paired sample t-test [42] was used to make an intramethodical comparison of the estimates of healthy and diseased kidneys as measured with ASL and DCE-MRI, respectively. Additionally, Bland-Altman plots [43] were drawn for both methods visualising the difference between healthy and diseased kidneys.

Further, to test the diagnostic significance regardless of absolute perfusion the relative perfusion of healthy and diseased kidneys, i.e. the ratios of cortical RBF were calculated and plotted.

## Results

**Figure 1:**
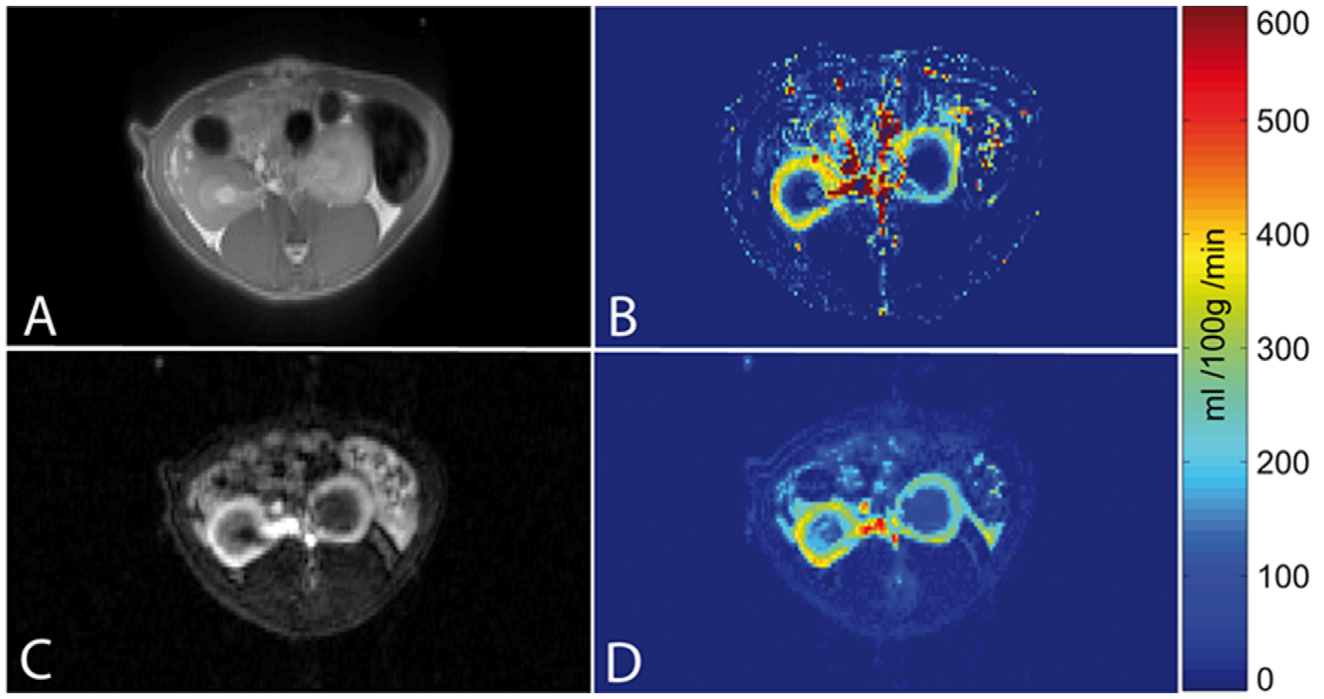
Exemplary illustration of perfusion MRI. Exemplary illustration of perfusion MRI of a rat with left-side AKI and perfusion maps. (**A**) True-FISP M_0_ image of an ASL measurement and the corresponding perfusion map (**B**). (**C**) TWIST post-contrast agent injection image and the corresponding RBF map (**D**). All drawings show the same rat and the same axial slice. Differences between the kidney with AKI and the contralateral kidney are clearly visible on the MRI images as well as on the perfusion maps.

**Figure 2:**
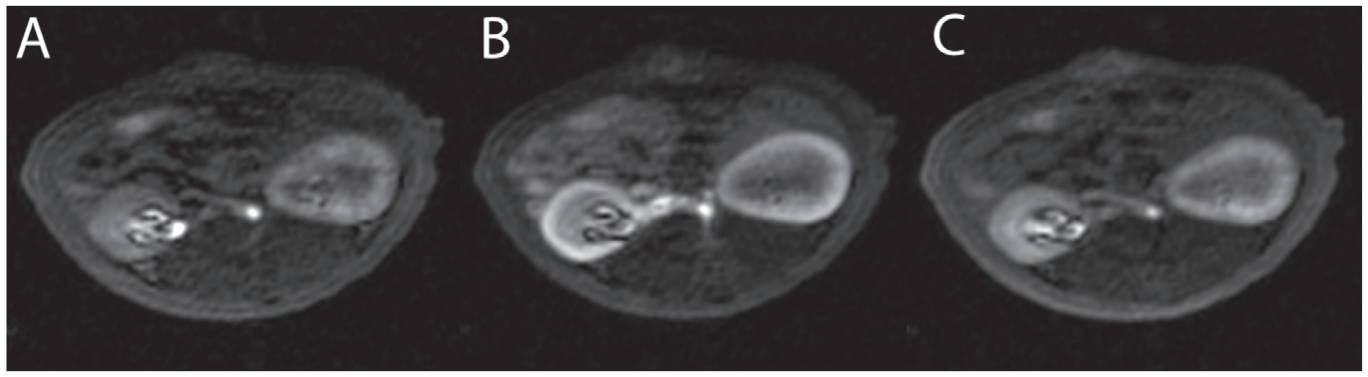
Representative DCE-MRI images. Representative DCE-MRI TWIST images at different points in time. (**A**) Baseline image before contrast agent injection. (**B**) Cortical peak contrast image approximately 40 s after bolus injection and an image (**C**) taken approximately 4 min later showing increased medullar contrast. Regarding the contrast uptake and its distribution, the AKI kidney can clearly be distinguished from the healthy kidney.

**Figure 3:**
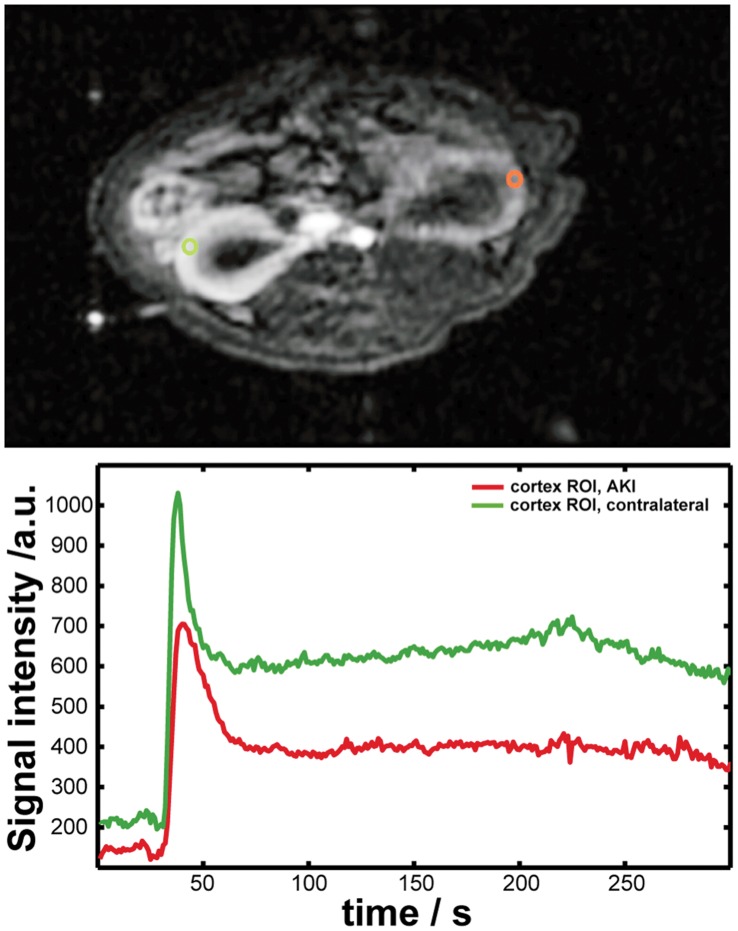
Time course of contrast uptake in DCE-MRI. The lower panel shows two exemplary DCE-MRI time courses of the tissue signal intensities (**B**), i.e. the contrast uptake in two cortical ROIs. In the upper panel (**A**) the position of the according ROIs is depicted. One ROI was placed in the healthy kidney (green), one in the diseased kidney (red). Please note that choice and shape of the ROIs in this figure are different from the ones drawn for the data evaluation.

**Figure 4:**
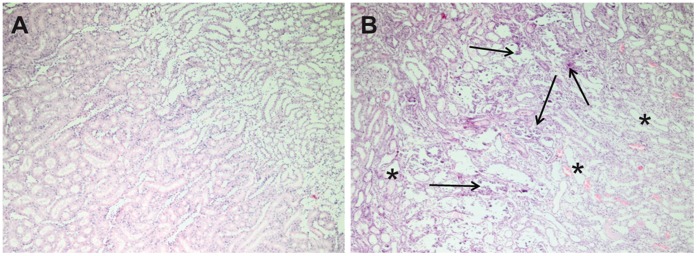
Histology of a native and a diseased kidney. Histological slides (H&E, 40x) of a native kidney (**A**) and a kidney five days after acute injury (**B**). Compared to the native kidney the diseased kidney shows main hallmarks of acute renal injury like cellular swelling, necrosis and tubular dilatation. Examples for necrosis and tubular dilatation are marked with arrows and stars, respectively.

The rats were examined without any incidents and ASL and DCE-MRI data sets were obtained from each rat. All recorded images showed diagnostic image quality and could be used to calculate perfusion maps. No image distortions or artefacts concerning the kidneys and possibly invalidating the quantitation, were visible. In both examinations the renal cortex could be outlined from the rest of the kidney. Figure 1 exemplarily shows one image of an ASL and a DCE-MRI examination of one rat, respectively. Additionally, the according RBF maps are presented. Figure 2 shows images of one representative DCE-MRI examination at three different phases. Shortly after the bolus injection the maximum contrast uptake in the renal cortex was reached. Four minutes after the injection, the filtration process of the kidneys led to equal signal intensities in cortex and medulla, however, the signal was still increased compared to the baseline image. The diseased kidney showed a decreased contrast uptake and the distinction between renal cortex and medulla was less pronounced, especially in the images after the first pass of the contrast agent. This can also be seen in Figure 3, which shows the time course of the signal intensities, i.e. the contrast uptake in two cortical ROIs. One ROI was placed in the healthy kidney, one in the diseased kidney. The decreased perfusion in the diseased kidney is clearly reflected by the decreased contrast uptake when compared to the healthy kidney. Generally, the diseased kidneys were increased in size. The changes of the kidneys with acute injury are particularly visible on histology. Figure 4 exemplarily shows the histology of a native kidney and a kidney five days after AKI. Compared to the native kidney the diseased kidney shows main hallmarks of AKI like cellular swelling, necrosis (arrows) and tubular dilatation (stars).

The results of the ASL and DCE-MRI examinations for the cortical RBF in both left and right kidney of each rat can be found in Table 1. We found the mean cortical RBF in the diseased kidneys to be 316±102 ml/100 g/min (ASL) and 407±119 ml/100 g/min (DCE-MRI). The mean cortical perfusion of the healthy kidneys was 416±124 ml/100 g/min (ASL) and 542±85 ml/100 g/min (DCE-MRI).

**Table 1:** Cortical RBF in ml/100 g/min as estimated from ASL and DCE-MRI measurements.

Rat	ASL		DCE	
	left (AKI)	right (healthy)	left (AKI)	right (healthy)
1*	(295)^1^	304	(535)^1^	519
2	456	634	321	433
3	191	344	313	481
4	289	504	566	631
5	374	462	504	679
6	269	371	330	517
Mean	*316±102*	*416±124*	*407±119*	*542±85*

* Animal with two healthy kidneys.

1 This value was included in the calculation of the mean RBF of the healthy kidneys and excluded when calculating the mean perfusion of the left (AKI) kidneys.

**Figure 5:**
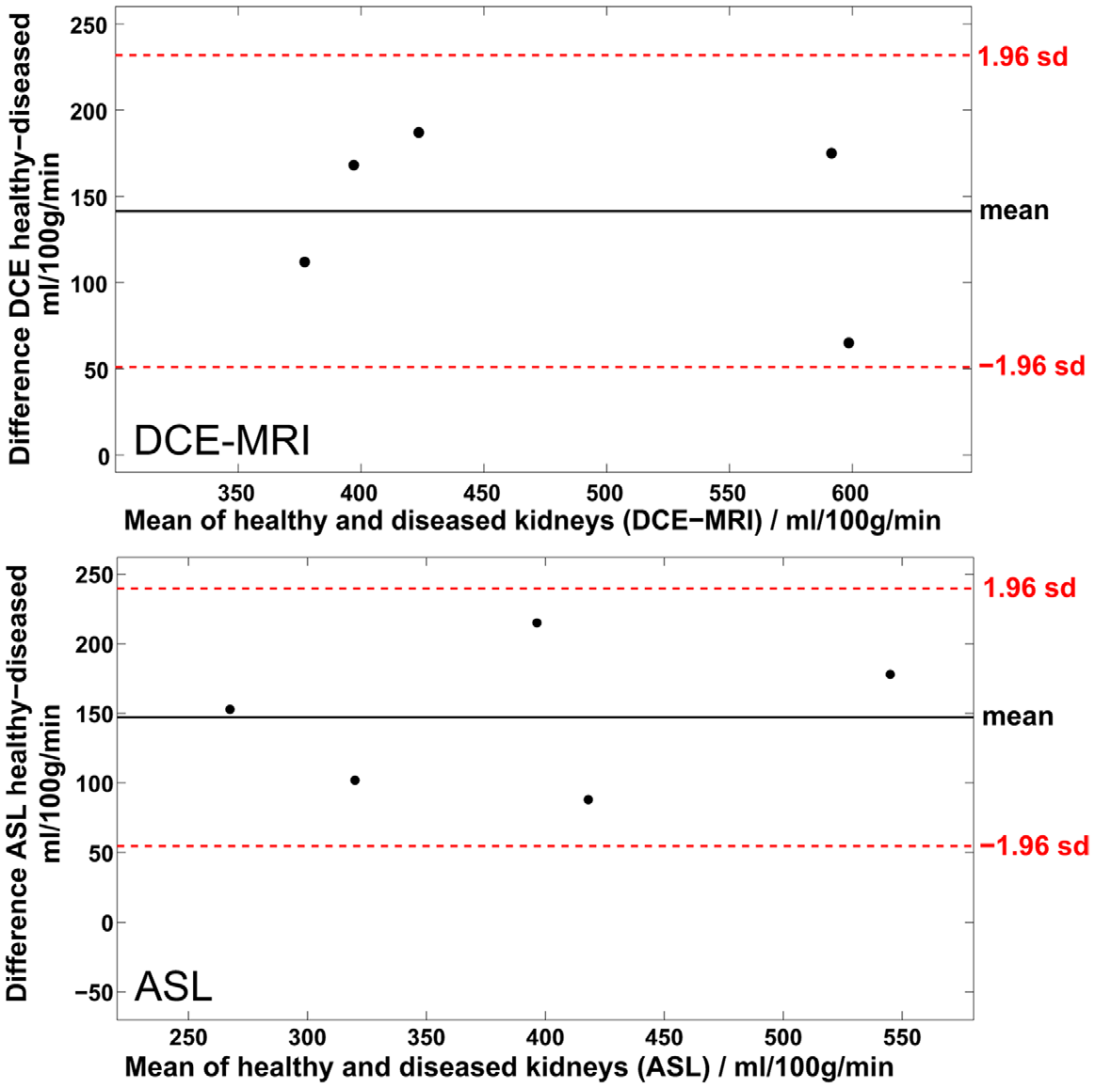
Comparison of renal perfusion between healthy and diseased kidneys. Differences in renal perfusion between healthy and diseased kidneys visualised in two Bland-Altman plots. The upper plot shows the differences as measured with DCE-MRI, the lower plot shows the differences as assessed with ASL. Both mean differences are significantly different from zero as zero is not included in the limits of agreement, i.e. the dotted lines that denote 1.96 times the standard deviation of the mean.

The paired sample t-test showed that the perfusion estimates of healthy and diseased kidneys are significantly different for both methods. The p-values of the t-test, i.e. the probability that the pairwise differences of RBF between healthy and diseased kidneys of one method are random variables from a normal distribution with mean zero and unknown variance, were computed to 0.34% and 0.36% when measured with ASL and DCE-MRI, respectively. This difference is also reflected in the Bland-Altman plots in Figure 5. Both plots show the systematically higher perfusion estimates for the healthy kidneys. The fact that the difference of zero is not included in the limits of agreement (±1.96 standard deviations of the average difference) strongly supports the ability of both methods to provide significantly different values for healthy and diseased kidneys. For ASL the mean RBF difference was 147±47 ml/100 g/min and for DCE-MRI 141±46 ml/100 g/min. The difference in RBF between healthy kidneys and kidneys with AKI is in very good agreement, whereas the variations of RBF between animals are slightly higher for the ASL estimates.

The results of the repeated ASL measurements can be found in Table 2. The small standard deviation in the RBF of both rats shows the small variability of the method within one subject and provides evidence for the robustness of the measurement.

**Table 2:** Perfusion estimates of repeated ASL measurements in two animals. All values are in ml/100 g/min.

Measurement Nr.	Rat 2		Rat 4	
	left(AKI)	right(healthy)	left(AKI)	right(healthy)
1	456	634	289	504
2	425	615	286	502
3	430	651	274	507
4	433	650	272	509
Mean	*436±12*	*638±15*	*280±7*	*506±3*

Regarding the comparison of RBF values between ASL and DCE-MRI, both estimates for each laterality, show systematically higher values for DCE-MRI. However, they are not significantly different.

**Figure 6:**
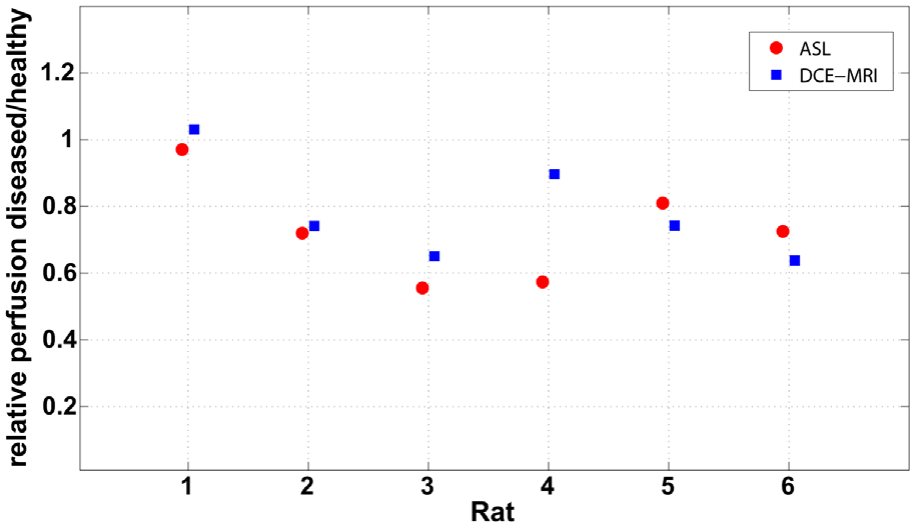
Relative perfusion between healthy and diseased kidneys. Graph of relative perfusion between AKI kidneys and healthy kidneys for each rat. The red circles represent the ratios as found with the ASL measurement. The blue squares depict the ratios as measured with DCE-MRI.

To compare ASL and DCE-MRI, regardless of absolute perfusion values, the RBF ratios between diseased and healthy kidneys are listed in Table 3 and visualised in Figure 6. Except for rat four the values are in excellent agreement. The rats with unilateral AKI can clearly be distinguished from the animal (rat one) with two healthy kidneys.

**Table 3:** Ratios of cortical RBF between left and right kidneys as estimated with ASL and DCE-MRI.

Rat	ASL	DCE
	RBF ratio left/right	RBF ratio left/right
1	0.97	1.03
2	0.72	0.74
3	0.56	0.65
4	0.57	0.90
5	0.81	0.74
6	0.73	0.64

## Discussion

This study showed that both ASL and DCE-MRI provide significantly (P<0.01) different values for the perfusion of healthy kidneys and kidneys with ischaemic AKI. This shows that both methods are capable of distinguishing the hypoperfusion of a kidney with AKI from the perfusion of a healthy kidney. We conclude that in future studies ASL can be sufficient to assess pathological changes in renal function by measuring renal perfusion. This is supported by the fact that the mean differences of renal cortical perfusion between healthy and diseased kidneys estimated by ASL and DCE-MRI are in very good agreement when averaging over all rats. A paired sample t-test showed no significant differences in the absolute estimates between the two methods. By performing repeated measurements in two animals we were able to show the robustness of the ASL RBF estimates. Table 3 shows that the variabilities within one animal are very small. This provides evidence for the validity of the results and the assumption of an underlying normal distribution of the animal population, despite the small sample size of only five animals with diseased kidneys. Unfortunately, it was not possible to repeat the DCE-MRI measurements as it would have taken too long for the animals to wait for a sufficient clearance of the contrast agent. The validity of the DCE-MRI estimates is however strengthened by the constant offset to the ASL estimates regarding the absolute RBF values (see Figure 5). However, in future studies it would be beneficial to increase the sample size.

So far, to our knowledge, this is the first study that uses an inter- and intramethodical comparison to test the significance and the comparability of two perfusion MR measurements in a rat model of AKI at a 3T whole body scanner. There are only two other studies [44], [45] that directly compare the estimates of quantified renal perfusion from ASL and DCE-MRI. However, both Wu et al. [45] and Winter et al. [44] solely assessed the perfusion of healthy kidneys which did not allow for an intramethodical test of diagnostic significance. Winter et al. measured the RBF of six rabbits with normal kidneys using a 1.5 T scanner. The study reports an average cortical RBF of 328±59 ml/100 g/min (ASL) and 357±96 ml/100 g/min (DCE-MRI). The ASL values are similar to our findings for the healthy kidneys (416±124 ml/100 g/min), however RBF as estimated with contrast-enhanced MRI is clearly lower than our findings (542±85 ml/100 g/min). Another difference to our study is that only the RBF of one, healthy kidney per animal has been acquired so that no comparison to the contralateral side was possible.

Wu et al. conducted a study on nineteen healthy humans and investigated the correlation between the two modalities. Similar to our results, they found the DCE-MRI values to be systematically higher and not entirely comparable to the ASL estimates.

Other recently published studies, where renal perfusion was measured in humans, state values close to our estimates. For example, ASL measurements using a similar FAIR True-FISP approach, report a cortical perfusion of 375±54 ml/100 g/min [16] and 376 ml/100 g/min [20]. When assessing the cortical RBF in humans with DCE-MRI by using a model-free deconvolution, Sourbron et al. [5] reported a value of 331±70 ml/100 g/min. This comparison to human perfusion studies is worth mentioning as the gained knowledge regarding technical aspects of the measurements can easily be transferred to assess renal RBF in humans.

A study that investigated the ambilateral RBF of rats with unilateral arterial occlusion (RAS) was performed by Pedersen et al. [46] who measured hemodynamic parameters of the kidney by dynamic susceptibility weighted (DSC) – MRI using ultra small superparamagnetic iron oxide (USPIO). Overall the data of 14 animals were acquired in that study and led to a mean cortical RBF of 431 ml/100 g/min and 410 ml/100 g/min in the healthy and RAS kidneys, respectively. Again, the RBF of the healthy kidneys is in good agreement with our ASL measurement.

Regarding our study, both methods detect the hypoperfusion of the injured kidney and show significantly different values for normal kidneys compared to diseased kidneys. However, except for one animal, the perfusion estimates in healthy and diseased kidneys are systematically higher for DCE-MRI. This is also reflected in the mean perfusion estimates when averaged over all animals. It is evident that in some animals the cortical perfusion of the diseased kidney is higher than the RBF in the healthy kidney of other rats, even when comparing values of only one method. Intramethodical variations are mainly attributed to different physiological conditions of the animals. Although the AKI was induced in the very same way each time, the rats and particularly the kidneys can respond differently to a certain degree which leads to a different extent of the injury. However, this can only explain variations in the differences in RBF between healthy and diseased kidneys. Another potential factor leading to intramethodical variations is the different impact of the anaesthesia during MRI on the animals and their kidney function. Especially, thiobutabarbital sodium can eventually alter the kidney function [47]. Also, the ad libitum approach regarding the fluid intake prior to the MRI experiment might result in different physiological states of the kidneys. In future studies, going without fluids for some hours prior to the experiment might be beneficial for consistency, although we think that these variations are minor compared to the ones mentioned above. Generally perfusion in small animals is sensitive to the body temperature. Although consecutive ASL measurements showed no changes in RBF our study is limited by the lack of temperature monitoring as absolute perfusion values might be influenced right after anaesthesia. However, the main focus of this study, the intra-animal comparison, is not affected.

An aspect that can lead to intra- as well as intermethodical variations is motion due to the respiratory cycle. This is a general problem when measuring in the abdomen. Although, motion was very weak in our case, it can generally lead to a blurring of the images which can invalidate the true contrast that is essential for the quantitation as it directly reflects the perfusion (ASL) and the tissue contrast uptake (DCE-MRI), respectively. Both imaging sequences acquire the central k-space lines first which assures that the image contrast is captured very rapidly. This lowers the motion sensitivity of both imaging sequences significantly but cannot fully eliminate it. For DCE-MRI a further reduction might be achieved by the usage of alternative k-space samplings like radial or BLADE [48] or through postprocessing using image registration techniques [49]. Unfortunately, FAIR ASL might not only be sensitive to motion during imaging but also to motion during the inflow time, i.e. the time between the labelling and the read-out. In such case, the perfusion signal can be invalidated when the imaging slice is shifted outside the ss-IR volume which leads to an overestimation of absolute perfusion. Regarding the observed motion and the fact that the ss-IR slice thickness was twice as thick as the imaging slice, we consider these errors to be negligible. In contrast to this, motion can also lead to a slice-selective inversion that labels the aorta at a point cranial to the branch of the renal arteries. This would result in an underestimation of RBF. Due to technical limitations, such an erroneous labelling of the aorta can anyway not be fully excluded and might explain the systematically lower ASL perfusion estimates. Remedy from motion corrupted ASL measurements can be found in respiratory triggering or navigators [20].

Another potential source for variations and errors can be partial volume effects. Regarding the slice thickness of the True-FISP read-out, such effects cannot be fully excluded. However, given the perpendicular orientation of the renal cortex with respect to the axial imaging slices and taking into account the high in-plane resolution, the impact on absolute quantitation should be negligible.

Compared to ASL, DCE-MRI has the advantage that functional parameters beyond perfusion can be extracted. However, quantitation of DCE-MRI heavily relies on a good selection of the AIF [50]. Slight variations can influence the outcome of the pharmacokinetic modelling and may contribute to inter-animal variation. Generally, it would be desirable to measure a local AIF. While this is already challenging in humans, it is nearly impossible in small animals. Therefore, the AIF was measured in the aorta to exclude partial volume effects due to an insufficient vessel size. The employed, well-standardised warm ischaemia model mainly damages the microvascular structure of the kidney as renal microcirculation is a key actor in the development of AKI [32]. Nevertheless, we cannot fully exclude errors in RBF quantification due to the dispersion of the AIF [51] on the path to the renal cortex. However, we expect these errors to be small and, if present, they should have no considerable effect on the identification of the hemodynamic abnormalities in the diseased kidney, i.e. the decreased perfusion compared to the healthy kidney. Especially, since the warm ischaemia model is known to decrease renal perfusion, the lower RBF will exceed potential underestimations in absolute perfusion caused by dispersion. This is also supported by the fact that the mean RBF differences between healthy and diseased kidney is identical for ASL and DCE-MRI. Furthermore, the exact vascular transit function is unknown and a local AIF is not accessible. Hence, an attempt to correct possible dispersion errors would very likely lead to an erroneous correction.

Generally, DCE-MRI benefits from a high temporal and spatial resolution as the sampling of the bolus curve is decisive and the possibility to easily distinguish different anatomical regions should be given. In our case, the temporal resolution is higher by a factor of four than the constraint demanded by Michaely et al. [52] for humans. This should sufficiently account for the faster blood circulation in rats.

The main advantage of ASL is the fact that it is completely non-invasive as water in arterial blood is used as an endogenous tracer which is especially interesting for patients where the injection of a Gd-based contrast agent is contraindicated. Therefore, it can generally be repeated as often as necessary. Also, ASL quantitation does not require an AIF because its signal is directly related to perfusion. However, it suffers from low SNR as the ASL signal is typically less than 1% of the tissue signal intensity. Hence, it is all the more beneficial to reduce any tissue signal as it is also a subtraction technique. Regarding this issue, our measurement could be improved by using background suppression [26] to zero the static tissue signal and thereby increase the relative perfusion signal. Another critical issue of pulsed ASL sequences is the ss-IR pulse and the quality of its inversion, regarding inversion accuracy and the shape of the inversion slab. Although the used inversion pulse showed a homogenous, complete inversion and an abrupt drop at the edges when applied in a water phantom, its inversion capabilities might be different in the abdomen due to air – tissue transitions that cause inhomogeneities in the magnetic field. An incomplete ss-IR can lead to increased perfusion estimates. Regarding the quantitation of ASL, it can be beneficial to measure a T1-map of the kidneys. This way the global T1 used for quantitation could be substituted by a value that reflects the longitudinal relaxation time in the according pixel itself. Especially, for the diseased kidney, T1 values of the renal cortex as found in the literature could be differing from the actual values.

In conclusion, this study showed that ASL and DCE-MRI are both capable of discriminating the hypoperfusion of a diseased kidney when compared to a healthy kidney. We demonstrated that both methods provide significantly different perfusion estimates between healthy and diseased kidneys. This qualifies both methods as tools to detect an acute kidney injury, as both provide the necessary sensitivity to detect the hypoperfusion caused by the ischaemic AKI. Absolute quantitation of cortical RBF in rats is not significantly different but DCE-MRI estimates are systematically higher. Nevertheless, it was shown that even when both methods differ in absolute RBF values, relative perfusion between left and right kidney can still indicate kidney diseases that are reflected in abnormal perfusion. For the diagnosis of renal diseases, this is the crucial requirement a method has to fulfil.

Microvascular perfusion of the renal cortex is an important parameter of high clinical value as it allows for directly drawing conclusions on the organ viability and function. Regarding the diagnostic significance, this study showed that ASL is a capable alternative to DCE-MRI, leaving it open to physicians to choose between two comparable methods. Especially for patients with impaired kidney function where the injection of Gd-based contrast agents may be contraindicated or for studies that involve drugs and diseased kidneys, ASL might be favoured. Nevertheless, regarding absolute perfusion values, nontrivial differences and variations remain when comparing the two methods, which indicate that further investigation and the comparison to a reference standard is needed.
